# Honeybees show an increased preference for dietary alcohol when parasitized

**DOI:** 10.1093/beheco/araf121

**Published:** 2025-10-29

**Authors:** Monika Ostap-Chec, Weronika Antoł, Daniel Bajorek, Daniel Stec, Krzysztof Miler

**Affiliations:** Doctoral School of Exact and Natural Sciences, Jagiellonian University, Prof. Stanisława Łojasiewicza 11, 30-348 Kraków, Poland; Institute of Environmental Sciences, Faculty of Biology, Jagiellonian University, Gronostajowa 7, 30-387 Kraków, Poland; Institute of Systematics and Evolution of Animals, Polish Academy of Sciences, Sławkowska 17, 31-016 Kraków, Poland; Institute of Systematics and Evolution of Animals, Polish Academy of Sciences, Sławkowska 17, 31-016 Kraków, Poland; Institute of Systematics and Evolution of Animals, Polish Academy of Sciences, Sławkowska 17, 31-016 Kraków, Poland; Institute of Systematics and Evolution of Animals, Polish Academy of Sciences, Sławkowska 17, 31-016 Kraków, Poland

**Keywords:** ethanol consumption, feeding behavior, *Nosema*, self-medication, *Vairimorpha*

## Abstract

Parasitic infections often alter host behavior, including foraging and the consumption of bioactive substances. In honeybees (*Apis mellifera*), infection with the common gut parasite *Nosema ceranae* causes metabolic disruption and increased mortality. Ethanol is a naturally occurring bioactive compound found in nectar, and honeybees exhibit high tolerance and resilience to chronic exposure. However, whether honeybees actively use ethanol during infection remains unclear. Here, we investigated whether *N. ceranae*-infected honeybees alter their ethanol consumption. In a feeding experiment, infected and uninfected honeybees were given a choice between plain sucrose solution and ethanol-spiked food (0.5% or 1% ethanol). We measured food consumption, survival, and spore load. Although overall food intake did not differ between groups, infected honeybees consumed a significantly higher proportion of ethanol-spiked food. Survival analysis showed that a diet containing 1% ethanol caused higher mortality than a diet containing 0.5% ethanol; however, among honeybees on a 1% ethanol diet, this negative effect was less pronounced in infected individuals than in controls. Spore load did not differ between treatments. These results suggest that *N. ceranae* infection induces a shift in feeding behavior toward increased ethanol intake, which may benefit infected honeybees by reducing mortality. This may reflect a self-medication response, although alternative explanations remain possible. Further research into ethanol's effects on *Nosema* spores is needed. Nonetheless, our findings provide insights into honeybee interactions with bioactive compounds and suggest that ethanol may be a behaviorally relevant dietary substance.

## Teaser

When animals become infected, they may use bioactive substances from their environment and adjust their diet to cope with disease. We investigated whether ethanol - a compound naturally occurring in nectar - plays such a role in honeybees, the world's key pollinators. We found that infected bees increased their ethanol intake, which was associated with improved survival. These results suggest that ethanol may have a behaviorally relevant function, potentially indicating self-medication in honeybees.

## Introduction

Parasites are among the most ubiquitous organisms on Earth, imposing significant fitness costs on their hosts (Dobson et al. 2008). Throughout coevolution, hosts have developed various defensive strategies to combat parasitic infections. Responses to parasites involve a range of behavioral adaptations, and combating endoparasites, particularly gut parasites, often requires some form of dietary changes. These strategies vary from alterations in food intake, diet preferences, or foraging behavior (Bernardo and Singer 2017; Schmid-Hempel 2021; Cotter and Al Shareefi 2022) to more advanced mechanisms, such as self-medication, in which infected individuals actively seek out substances that, although harmful to healthy organisms, possess therapeutic properties (de Roode et al. 2013; Abbott 2014). Such behavioral responses are not always adaptive; they may also result from parasite manipulation or be unintended by-products of the infection (Vale et al. 2018). Dietary changes influenced by parasitic infection have been documented across various taxa, including insects. For instance, infected caterpillars opt for diets higher in protein and lower in carbohydrates, which increases their chances of survival (Povey et al. 2014). Honeybees actively select foods with antibiotic properties when infected (Gherman et al. 2014; Pusceddu et al. 2019). Infected ant colonies have also been observed to choose a diet that, while detrimental to long-term survival, provides short-term benefits in fighting infection (Csata et al. 2024).

Honeybees are one of the most important pollinators for both natural ecosystems and agriculture (Klein et al. 2018; Papa et al. 2022) and face various parasites. *Nosema ceranae* and Nosema *apis* are two extensively studied microsporidia due to their commonness and significant impact on honeybee health (Moritz et al. 2010; Hristov et al. 2020; Ostap-Chec et al. 2024a). In the external environment, they exist as spores, which must be eaten by a bee, either through food or water, for the infection to initiate (Fries 2010; Smith 2012). Spores can also be transferred sexually between drones and queens (Roberts et al. 2015). When the spores reach the midgut, they invade the epithelial cells and rapidly multiply, consuming the cell's contents through phagocytosis and depleting the host's resources (Gisder et al. 2011). The spores released upon cell destruction can infect other cells or be expelled, contaminating floral resources and the nesting environment. Over time, this cell destruction leads to gut lesions, impaired nutrient absorption, and other detrimental effects (Goblirsch 2018; Paris et al. 2018).

Of these two *Nosema* species, *N. ceranae* has garnered more attention due to its wider host range, ability to infect year-round with minimal seasonal variation, and higher biotic potential across different temperatures (Martín-Hernández et al. 2009; Higes et al. 2010). Moreover, it is globally distributed and has been reported on all continents (Grupe and Quandt 2020). Unlike *N. apis*, *N. ceranae* does not cause conspicuous disease symptoms such as dysentery. However, infected honeybees still suffer from digestive disorders, lethargy, and a shortened lifespan (Higes et al. 2008, 2009, 2010; Botías et al. 2013; Koch et al. 2017; Ostap-Chec et al. 2024a). *N. ceranae* infection disrupts energy metabolism in honeybees, as evidenced by the dysregulated expression of metabolic genes (Vidau et al. 2014; Kurze et al. 2016). This metabolic stress is accompanied by significant immunosuppression (Antúnez et al. 2009; Chaimanee et al. 2012; Holt et al. 2013; Huang et al. 2016; Li et al. 2018; Lourenço et al. 2021). Several studies have reported a decline in hemolymph trehalose—a key energy-storage sugar—in infected honeybees (Blatt and Roces 2001; Thompson 2003; Mayack and Naug 2010; but see Ostap-Chec et al. 2025b). Infected honeybees demonstrate increased respirometric activity and lipid loss (Li et al. 2018), ultimately leading to fat body depletion (Gilbert et al. 2024). These metabolic disruptions are further facilitated by the fact that *Nosema* spores lack mitochondria and rely entirely on the host for essential energy resources (Tsaousis et al. 2008). Such energetic strains manifest not only in physiological impairments, including disrupted digestion, thermoregulation, and increased susceptibility to starvation (Mayack and Naug 2009; Martín-Hernández et al. 2011; Vidau et al. 2014), but also in behavioral changes, such as prolonged foraging times and reduced flight frequency (Dussaubat et al. 2013; Alaux et al. 2014; Naug 2014; Wolf et al. 2014). Within colonies, *N. ceranae* prevalence increases with age—being rarely detected in newly emerged bees, moderate in nurses, and highest in foragers (Smart and Sheppard 2012; Jack et al. 2016; Li et al. 2017). As foragers are responsible for securing food stores, the disease ultimately affects the whole colony and reduces its overall success (Emsen et al. 2020).

How honeybees combat *Nosema* infections remains insufficiently studied. Behavioral avoidance appears particularly challenging, as the disease primarily spreads through fecal-oral transmission and contaminated floral resources (Fries 2010; Smith 2012). Studies show that infected honeybees exhibit precocious and increased flight activity, which may help reduce pathogen transmission while keeping healthy honeybees engaged in safer hive tasks and reducing pathogen transmission inside the hive (Dussaubat et al. 2013). Moreover, *Nosema*-infected honeybees prefer honey with higher antibiotic activity, which lowers microsporidian loads (Gherman et al. 2014). Surprisingly, despite the significant energetic stress caused by *Nosema* infection, honeybees do not compensate for these losses by increasing sucrose consumption (Ostap-Chec et al. 2025b). Propolis, a natural hive component, is known to reduce mortality, infection rates, and pathogen infectivity in *Nosema*-infected honeybees (Naree et al. 2021a, 2021b). However, there is no evidence that honeybees actively employ it to fight *Nosema* (Mura et al. 2020).

Ethanol is another naturally occurring substance that may serve as a potential medicinal compound. It is produced by yeast (such as *Saccharomyces cerevisiae*) during sugar fermentation, a process ongoing for around 100 million years (Thomson et al. 2005). Given the global abundance of flowering plants (Govaerts et al. 2021), and the frequent presence of yeasts in nectar (Lievens et al. 2015), ethanol occurs naturally in many ecosystems. While tropical flower nectars may contain up to 6.9% ethanol (Goodrich et al. 2006; Hockings et al. 2015), in temperate conditions concentrations rarely exceed 1% (Ehlers and Olesen 1997; Jakubska 2005; Goodrich et al. 2006; Wiens et al. 2008; Rering et al. 2018). Ethanol seems to be ecologically relevant and has likely shaped nutrition, behavior, and disease resistance in frugivorous vertebrates and nectarivorous invertebrates (Bouchebti et al. 2024; Bowland et al. 2025; Miler 2025). In honeybees, ethanol is a precursor of ethyl oleate, a pheromone regulating colony demography (Leoncini et al. 2004; Castillo et al. 2012a, 2012b). Moreover, foragers, produce alcohol dehydrogenase possibly to metabolize ethanol (Miler et al. 2022, 2021b).

Honeybees willingly consume ethanol dissolved in sucrose under both laboratory and field conditions, even at high concentrations reaching up to 20% (Abramson et al. 2000, 2004b; Maze et al. 2006; Sokolowski et al. 2012; Mustard et al. 2019). Moreover, foragers prefer feeding solutions containing up to about 2.5% ethanol over pure sucrose solutions (Mustard et al. 2019). Although the effects of ethanol on honeybees have been the subject of several studies, most of this research focused on single ethanol exposure, often using high, ecologically irrelevant doses. These studies have shown that such acute consumption impairs honeybee physiology and behavior. Specifically, ethanol disrupts social communication among workers by impairing antennation and trophallaxis (Mixson et al. 2010; Wright et al. 2012), alters dance communication (Bozic et al. 2006), and increases aggression (Abramson et al. 2004a; Ammons and Hunt 2008). Additionally, it negatively affects locomotion, foraging efficiency, and learning abilities (Abramson et al. 2005; Maze et al. 2006; Mustard et al. 2008; Giannoni-Guzmán et al. 2014; Black et al. 2021; Ahmed et al. 2022), with the severity of these impairments increasing in a dose-dependent manner (Bozic et al. 2006; Maze et al. 2006; Wright et al. 2012). In natural conditions, it is more realistic that bees encounter ethanol in relatively low concentrations but repeatedly. Studies that simulate such conditions show that honeybees exhibit tolerance, showing reduced motor impairment compared to naive individuals (Miler et al. 2018; but see Stephenson et al. 2021). Chronic dietary ethanol exposure, whether through voluntary consumption of 0.5% ethanol or forced intake of 1%, has no significant effect on survival, flight endurance, body mass, or lipid content, except for an increased trehalose level in the hemolymph (Ostap-Chec et al. 2025a). These findings suggest that honeybees are highly resilient to chronic intake of ethanol. However, research on older honeybees indicates that both occasional and continuous ethanol exposure significantly reduce survival, suggesting that the toxic effects of even low concentrations of ethanol may be more pronounced in aged individuals (Ostap-Chec et al. 2024b). Interestingly, honeybees subjected to prolonged ethanol consumption exhibit withdrawal symptoms upon discontinuation of access to ethanol-spiked food (Ostap-Chec et al. 2021), a hallmark characteristic of alcohol dependence.

Ethanol is a substance that, while detrimental in high doses, may have protective or otherwise beneficial effects at low concentrations. There are examples in the animal kingdom where ethanol is used for self-medication or to enhance reproductive success. *Drosophila melanogaster* preferentially oviposits on ethanol-containing substrates, which enhance offspring fitness (Bokor and Pecsenye 2000; Azanchi et al. 2013). Additionally, *D. melanogaster* larvae increase ethanol intake when parasitized by endoparasitoid wasps, suggesting a form of self-medication (Milan et al. 2012). A comparable ethanol-based mechanism has been observed in ambrosia beetles (*Xylosandrus germanus*), which rely on ethanol-rich environments to cultivate fungal gardens essential for their reproduction (Ranger et al. 2018). As ethanol is also a caloric substance, it may serve as an alternative energy source. Butterflies appear to be physiologically adapted to ethanol, as low concentrations do not impair their fitness traits (Miller 1997). They have evolved tolerance mechanisms allowing them to derive comparable energetic value from ethanol as from sugar, which may support fecundity in conditions of limited sugar availability (Beaulieu et al. 2017). A similar strategy is observed in the desert-adapted *Drosophila mojavensis*, where exposure to ethanol vapor increases longevity and fecundity under carbohydrate-poor conditions, suggesting that ethanol can function as an alternative energy source (Etges and Klassen 1989). This effect is genotype- and environment-dependent and likely reflects local adaptations (Starmer et al. 1977). Nevertheless, despite the frequent environmental exposure of honeybees to ethanol, its potential adaptive significance—whether medicinal, protective, or metabolic—remains insufficiently studied.

Here, we tested the hypothesis that honeybees alter their ethanol intake when infected with *N. ceranae*. This addresses key questions in behavioral ecology, such as whether parasitic infection induces changes in host feeding behavior, and whether such responses are adaptive (eg, self-medication) or not. We conducted a feeding experiment that mimicked a naturalistic choice scenario between ecologically relevant, low ethanol concentrations. Healthy and *Nosema*-infected honeybees were offered a choice between plain sucrose solution and either 0.5% or 1% ethanol-spiked sucrose, and we measured food consumption, survival, and spore load. Specifically, we asked whether (1) infected honeybees increase their intake of ethanol-spiked food compared to uninfected bees, (2) ethanol consumption alters mortality depending on infection status, and (3) parasite loads differ between ethanol concentrations. Based on prior evidence of high ethanol resilience in honeybees (Ostap-Chec et al. 2025a) and infection-induced changes in dietary preferences in other insect species (Milan et al. 2012; Gherman et al. 2014; Csata et al. 2024), we predicted an increase in ethanol consumption among infected bees, and a possible reduction in infection-related costs (eg, mortality and/or spore load) due to this dietary shift.

## Methods

### Experimental procedure

The experiment was conducted using queen-right colonies of the Western honeybees (*Apis mellifera carnica*) with naturally inseminated queens. The colonies were in good overall condition (with food reserves, brood, and relatively large populations) and had been treated with oxalic acid against *Varroa destructor* in early spring. Additionally, all colonies were screened for background *Nosema* infection (see below).

The study was performed in four replicates, with a 2-day interval between each replicate. For each replicate, newly emerged bees were obtained from two unrelated colonies. To achieve this, a brood frame with capped cells, free of adult bees, was collected from each colony and placed overnight in an incubator (KB53, Binder, Germany) set at 32 °C. In the following morning, all newly emerged bees were marked with a colored dot on the thorax using a non-toxic paint marker and introduced into an unrelated hive for 7 days. This step allowed the bees to develop in a natural hive environment, supporting physiological development, enhancing immune function, and increasing survival rates in subsequent laboratory conditions (Ostap-Chec et al. 2021, 2024b).

At 7 days old, the marked bees were recollected from the hive using entomological forceps, transferred to wooden cages, and transported to the laboratory for individual feeding. To enhance feeding motivation, the bees were food-deprived for approximately 1 h before the feeding procedure. Each bee was then placed in a plastic Petri dish with a lid (diameter 5.5 cm) and assigned to one of two groups: infected or control. Bees in the infected group received a 10 µl droplet of 1 M sucrose solution containing 100,000 *N. ceranae* spores (for details on solution preparation see below), while control bees received a 10 µl droplet of 1 M sucrose solution without spores. The bees were observed for up to 3 h, and only those that fully consumed their respective solutions were included in the experiment.

For each replicate, 20 cages were established, 10 for infected bees and 10 for control bees, each containing 40 individuals. Over the following 2 days, all bees received a 40% sucrose solution (Polski Cukier, Poland) and water, provided ad libitum via gravity feeders. The cages were maintained in an incubator (KB400, Binder, Germany) at 32 °C. After this acclimation period, the initial number of live bees in each cage was recorded. This period allowed the bees to recover from handling stress, stabilize their feeding behavior, and minimize bias from initial mortality.

After acclimation, bees within each group (infected vs. control) entered the experimental phase and were assigned to one of two dietary treatments. In each diet honeybees were provided with two feeders: one containing pure 1 M sucrose solution and the other containing sucrose solution with either 0.5% ethanol [diet: 0.5% EtOH] or 1% ethanol [diet: 1% EtOH]. Each dietary treatment was replicated in five cages per group. The ethanol-supplemented solutions were prepared to be iso-caloric with the 1 M sucrose solution by reducing the sucrose content by 1.38 g for each mL of ethanol (assuming 5.5 kcal/1 ml of ethanol) to prevent confounding effects related to ethanol's caloric value when digested (Ostap-Chec et al. 2024bMiler et al. 2022).

The bees remained on their assigned diets for 15 days. This period reflects the upper-level longevity of forager honeybees under natural conditions and thus corresponds to the typical duration of potential ethanol exposure in the wild (Dukas 2008). Every 3 days, both feeders in each cage were weighed before and after refilling to assess daily food consumption. As a result, food consumption was measured at five timepoints (on Days 3, 6, 9, 12, and 15 of the experimental phase, corresponding to Days 12, 15, 18, 21, and 24 of the bees' life). Mortality was recorded daily, and dead individuals were removed. In total, 3,200 bees were used in the experiment (4 replicates × 2 groups × 2 diets × 5 cages × 40 individuals). On the final day of the experimental phase, samples were frozen for molecular analysis to confirm infection status (infected vs. control).

### Preparation of spores for infection

We sourced the spores from our stock population of infected honeybees, which were maintained in controlled incubator conditions to sustain the infection for spore harvesting. The identity of *N. ceranae* spores in the stock population was confirmed as described by Berbeć et al. (2022). The spore suspension used for experimental inoculation was freshly prepared on the same day the bees were fed. To prepare the suspension, we homogenized the digestive tracts of several infected individuals using a micropestle in distilled water. The mixture was centrifuged (Frontier 5306, Ohaus, Switzerland) at 6,000 G for 5 min, repeating this process three times. After each centrifugation, the supernatant was replaced with fresh distilled water.

The final supernatant was replaced with a 1 M sucrose solution, and the concentration of spores was determined using a Bürker hemocytometer under a Leica DMLB light microscope equipped with phase contrast and a digital camera. The final infection solution was adjusted to achieve a concentration of 100,000 spores per 10 µl by diluting with 1 M sucrose solution.

### Verification of Nosema infection status

To confirm that bees exposed to *N. ceranae* spores were indeed infected and that bees in the control group remained uninfected, we assessed spore levels using qPCR quantification, according to the protocol of Antoł and Ostap-Chec (2025). We sampled 10 infected and 10 control bees per colony and dietary treatment (160 individuals in total).

Additionally, to ensure that the colonies used for the experiment were initially free from *N. ceranae* infection, we tested their infection status. For this, we collected two bees from the same brood frames used to gather 1-day-old bees (eight individuals in total), froze them, and subsequently analyzed their spore levels using qPCR.

### DNA extraction and sample preparation for qPCR

For DNA extraction, the abdomens of individual bees were cut using sterile instruments and placed into 2 ml cryovials, each containing 800 µl dH_2_O and ceramic beads (Omni International). The samples were homogenized using a Bead Ruptor ELITE homogenizer (Omni International). From each homogenate, a 200 µl aliquot was taken for DNA extraction following a solution-based protocol with Nuclei Lysis Solution and Protein Precipitation Solution (Promega). The resulting DNA pellets were resuspended in 100 µl of 1× TE buffer for further analysis. Extraction blanks (*N* = 7) were included in each DNA extraction batch to account for possible cross-contamination at this stage of sample processing.

### Quantification of *N. ceranae* load by qPCR

To quantify the parasite load, we amplified a 65-bp fragment of the *N. ceranae Hsp70* gene using primers designed by Cilia et al. (2018). Since the *Hsp70* gene exists as a single copy per spore, the measured copy number can be directly translated into the spore load per individual (Cilia et al. 2018, 2020).

The original DNA extracts were diluted 10× for qPCR which was performed using a CFX96 Touch Real-Time PCR Detection System (Bio-Rad) in a 20 µl reaction mix containing: 5 µl of DNA template, 10 µl of SsoAdvanced™ Universal SYBR® Green Supermix (Bio-Rad), target primers at a final concentration of 0.2 µM (see Table 1 for primer sequences), and ddH_2_O. The cycling conditions were as follows: an initial denaturation at 98 °C for 3 min, followed by 40 cycles of 98 °C for 15 s and 60 °C for 30 s.

**Table 1.** Primers used in qPCR for *Hsp70* amplification and absolute quantification.

Hsp70 target length	Primer	Primer sequence (5′ to 3′)	Reference
**65 bp (target)**	Cilia_Hsp70_F	GGGATTACAAGTGCTTAGAGTGATT	Cilia et al. (2018, 2020)
	Cilia_Hsp70_R	TGTCAAGCCCATAAGCAAGTG	
**824 bp (standard)**	Hsp70_broad_F	TGCGTCTAAGAGATTGCTGGG	Ostap-Chec et al. (2025b)
	Hsp70_broad_R	GCATTCGTGTCATTCCACCC	

For absolute quantification, a purified PCR product of a broader 824 bp *Hsp70* fragment was used as a standard. The fragment was amplified using previously designed primers and cycling conditions (Ostap-Chec et al. 2025b, see Table 1).

A standard curve for each plate was prepared using the purified product, with a dilution series covering a dynamic range of 8 logs (from 6.20 × 10^−1^ to 6.20 × 10^6^ copies). Standards were run in triplicate and samples in duplicate on each qPCR plate. The method demonstrated a sensitivity of 6.20 × 10^−1^ copies/µl (equivalent to 2,480 copies/bee or 3.39 log copies/bee), which was the lowest concentration in the dilution series with high reproducibility and strong linearity (*R*^2^ ≥ 0.98). PCR efficiencies for the standard curve between 90% and 110% were accepted. To confirm specificity, a melting curve analysis was performed at the end of each run, covering the temperature range of 65 to 95 °C in 0.5 °C increments, with a dwell time of 5 s per step.

### Calculation of spore load

To determine the spore load, the mean starting quantity of the template for each duplicate based on the standard curve was calculated. The results were expressed as the number of *Hsp70* copies/µl, which corresponds to the number of spores (following Cilia et al. 2018). Finally, the values were recalculated to the original homogenate volume and log_10_-transformed to obtain the spore load as the log number of spores per bee.

### Statistics

All statistical analyses were performed in R (R Core Team 2024). Daily food consumption at each timepoint was calculated as the sum of intake from both feeders (one containing pure sucrose and the other containing sucrose with either 0.5% or 1% ethanol), divided by the number of live bees in the cage over these 24 h. This value was expressed as per capita consumption (mg/bee). To analyze food consumption over time, we fitted a generalized additive mixed model (GAMM) using the *gamm* function from the *mgcv* package (Wood 2017, 2023), as this approach is well-suited to capture expected non-linear patterns (Heit et al. 2024). The model assumed a Gaussian distribution with an identity link function and included group (control vs. infected), diet (0.5% EtOH vs. 1% EtOH), and their interaction as fixed effects. To account for potential nonlinear trends in consumption over time, a smooth term for timepoint was included, with separate smoothing functions for each group-diet combination. The smooth terms were modeled using penalized regression splines with five basis functions (*k* = 5). Random effects for colony and cage nested within colony were included. The results were visualized using the *ggplot2* package (Wickham 2016).

The proportion of ethanol-spiked food consumed per bee at each timepoint was calculated as the intake from the ethanol-spiked feeder per bee divided by the total food consumed per bee. This proportion ranged from 0 (no ethanol-spiked food consumed) to 1 (only ethanol-spiked food consumed). To assess changes in the proportion of ethanol-spiked food consumed over time, we fitted a Generalized Linear Mixed Model (GLMM) using the *glmmTMB* function from the *glmmTMB* package (Brooks et al. 2017). The model assumed a beta distribution with a logit link function, which is appropriate for proportion data bounded between 0 and 1. Fixed effects included timepoint (1 to 5), group (control vs. infected), diet (0.5% EtOH vs. 1% EtOH), and their two-way interactions. The third-way interaction was removed as it was non-significant (*P* = 0.317), and its removal improved model performance based on AIC comparison. Random effects for colony and cage nested within colony were included. In addition, a dispersion formula was incorporated to adjust for overdispersion. To evaluate the significance of fixed effects, we used Type III Wald *χ*² tests as implemented in the Anova() function from the *car* package. The results were visualized using *ggplot2* (Wickham 2016).

For mortality analysis, we used Cox mixed-effects regression with the *survival* package (Therneau 2022, 2023). The model included group (control vs. infected), diet (0.5% EtOH vs. 1% EtOH), and their interaction as fixed effects, with colony and cage nested within the colony as random effects. To follow up the significant interaction, we used the *emmeans* package (Lenth 2025) to perform post hoc pairwise comparisons of diet effects within each group, using contrasts on the response scale. Survival curves were visualized using *ggsurvplot* from the *survminer* package (Kassambara et al. 2021).

The effect of dietary treatment on *N. ceranae* spore load in the infected group (*N* = 80) was analyzed using linear quantile mixed model, estimating median (*lqmm* function from the *lqmm* package (Geraci 2014)), with log mean quantity of *Nosema* spores per bee as response variable, diet as fixed effect, and combination of colony and cage as random effect. The spore load was visualized using *ggplot2* (Wickham 2016).

## Results

**Fig. 1.**
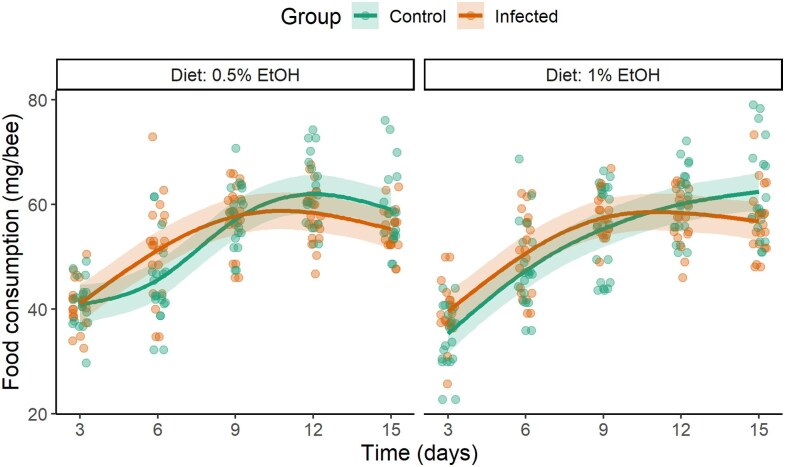
Per capita food consumption (mg/bee) in control and infected bees across five timepoints (measured every 3 days during 15 days) for two dietary treatments (0.5% EtOH diet vs. 1% EtOH diet). No significant differences were observed either between groups or between diets. Data points represent raw individual observations, while lines indicate GAMM predictions, and shaded areas indicate 95% confidence intervals.

The analysis revealed that neither group (control vs. infected) nor diet (0.5% EtOH vs. 1% EtOH) had a significant effect on total food consumption in honeybees (group: estimate ± SE = −0.178 ± 0.856, *t* = −0.208, *P* = 0.835; diet: estimate ± SE = −0.842 ± 0.856, *t* = −0.984, *P* = 0.326; interaction: estimate ± SE = 0.6577 ± 1.210, *t* = 0.544, *P* = 0.587). Food consumption followed a significantly non-linear pattern across all group-diet combinations (*P* < 0.001 for all smooth terms; Fig. 1). The degree of nonlinearity differed among combinations, with the highest non-linearity in the control group under the 0.5% ethanol diet (edf = 3.47, *F* = 52.69, *P* < 0.001) and the lowest in the control group under the 1% ethanol diet (edf = 2.73, *F* = 97.34, *P* < 0.001). In all cases, food consumption increased during the first three timepoints and then stabilized. The model explained 58.5% of the variance (*R*² = 0.585).

**Fig. 2.**
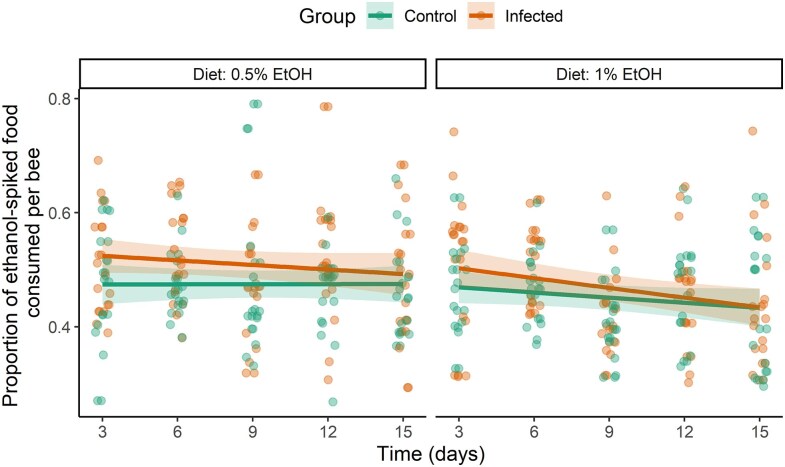
Proportion of ethanol-spiked food consumed by bees from control and infected group across five timepoints (measured every 3 days during 15 days) under two dietary treatments (0.5% EtOH diet vs. 1% EtOH diet). The proportion of consumed ethanol-spiked food was significantly higher in infected bees compared to controls. Data points represent raw individual observations, while lines represent GLMM predictions, and shaded areas indicate 95% confidence intervals.

Infection had a significant effect on the proportion of ethanol-spiked food consumed by honeybees (estimate ± SE = 0.233 ± 0.096, *z* = 2.433, *P* = 0.015; Fig. 2). Infected bees had 26% (4.64% to 52.30%) higher odds of consuming ethanol-spiked food compared to the control group. Other factors and interactions were non-significant (Table 2).

**Table 2.** Results of the GLMM showing Type III Wald *χ*² tests for effects of timepoint, group (control vs. infected), diet (0.5% EtOH diet vs. 1% EtOH diet), and their two-way interactions on the proportion of ethanol-spiked food consumed by honeybees.

	*χ* ^2^	Pr(>Chisq)
**Timepoint**	0.001	0.978
**Group**	5.919	0.015*
**Diet**	0.030	0.863
**Timepoint × group**	1.446	0.229
**Timepoint × diet**	1.849	0.174
**Group × diet**	0.764	0.382

Results were considered significant at *P* < 0.05.

**Fig. 3.**
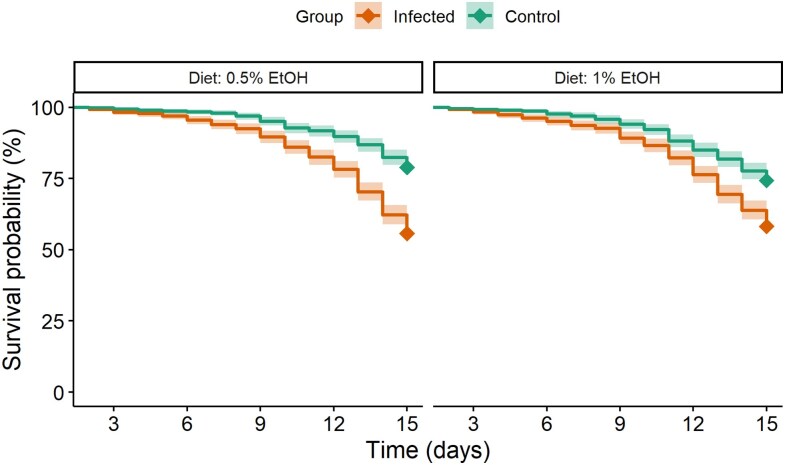
Survival plot for control and *Nosema*-infected bees over the experiment. For each replicate, 10 cages were assigned to infected bees and 10 to control bees, with five cages per diet. Each cage started with 37 to 40 bees. Lines represent Cox regression predictions, and shaded areas show 95% confidence intervals.

Mortality rates differed significantly between groups and diets (Fig. 3). Infected bees had a 2.49-fold higher risk of death than controls (hazard ratio = 2.49, SE = 0.094, *z* = 9.70, *P* < 0.001). Bees on the 1% ethanol diet had a 26% higher risk of death compared to those on the 0.5% ethanol diet (hazard ratio = 1.26, SE = 0.104, *z* = 2.23, *P* = 0.026). Notably, the significant interaction between group and diet (hazard ratio = 0.75, SE: 0.130, *z* = −2.17, *P* = 0.030) indicates that the negative effect of 1% ethanol on survival was weaker in infected bees than in controls. Post-hoc comparisons confirmed that 1% ethanol significantly reduced survival compared to 0.5% ethanol in control bees (hazard ratio = 0.79, SE = 0.083, *z* = −2.23, *P* = 0.0258) but not in infected individuals (hazard ratio = 1.05, SE = 0.081, *z* = 0.63, *P* = 0.5260).

*Nosema* spore load (mean of the log mean quantity per individual ± SD) in the infected group (*N* = 80) was 6.46 ± 1.39, over 3 orders of magnitude higher than in the control group (*N* = 80, 3.11 ± 0.92), while the latter was comparable to background spore counts in bees sampled directly from the frames before the experiment (*N* = 8, 3.03 ± 0.38). Spore level in extraction blanks, recalculated to the same volume as experimental samples, was 2.43 ± 1.15. The spore levels in blanks, pre-experimental bees from frames, and control group bees were negligible and consistent with previous studies (Baffoni et al. 2016; Braglia et al. 2021; Garrido et al. 2024; Ostap-Chec et al. 2025b).

**Fig. 4.**
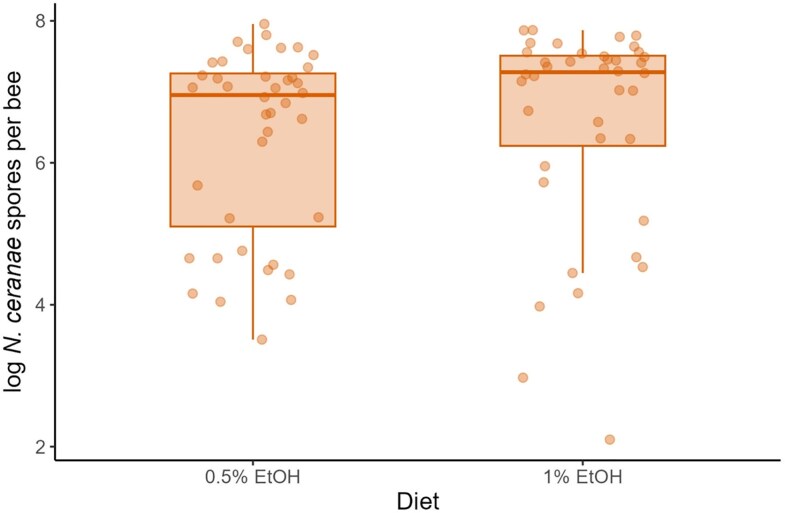
*N. ceranae* spore load per individual in the infected group for different diet treatments (0.5% EtOH vs. 1% EtOH). A single dot represents a single individual. Boxes indicate median and interquartile range, whiskers show 1.5 of the interquartile range.

Comparison of *Nosema* spore load for dietary treatments in the infected group (Fig. 4) showed no significant effect of the diet (log mean quantity estimated effect ± SE = 0.3355 ± 0.2497, *P* = 0.19).

## Discussion

The results of total food consumption indicate no difference between *Nosema*-infected and control honeybees. This aligns with our previous experimental and meta-analytic findings showing that *Nosema* infection does not affect overall carbohydrate intake (Ostap-Chec et al. 2025b), supporting the idea that infected honeybees do not compensate for energy loss through increased sugar consumption. It is important to emphasize, however, that under natural conditions, honeybees have access to a more diverse diet. Energetic stress in *Nosema*-infected honeybees likely involves not only altered sugar metabolism but also disruptions in protein metabolism and substantial lipid depletion (Aliferis et al. 2012; Badaoui et al. 2017; Li et al. 2018, 2019), with lipids potentially serving as an alternative energy source during infection. Nevertheless, our findings reinforce the conclusion that, at least in laboratory conditions, carbohydrate intake remains unchanged during infection, despite increased energetic demands (Mayack and Naug 2009, 2010; Holt et al. 2013).

Moreover, honeybees did not differ in total food consumption regardless of whether the diet contained 0.5% or 1% ethanol. Since our ethanol-containing solutions were prepared to be iso-caloric—meaning that both pure sucrose solutions and those supplemented with ethanol had the same energy content—we can confidently state that honeybees on these two diets did not differ in terms of appetite. Previous studies support this conclusion. Mustard et al. (2019) found that during a 24-h assay, honeybees given access to both ethanol-spiked (0.625% to 2.5%) and pure sucrose solutions consumed a similar total volume of food as control honeybees presented with two pure sucrose feeders. Food intake was significantly reduced only at ethanol concentrations of 5% and above. Similar patterns have been observed in other species: studies on rats and birds have shown that while high doses of ethanol can suppress appetite, low-level ethanol consumption does not affect food intake (Mazeh et al. 2008; Nelson et al. 2016). A comparable response was observed in Egyptian fruit bats (*Rousettus aegyptiacus*), which increased consumption at 0.1% ethanol, showed no effect on food intake at intermediate concentrations (0.3% to 0.5%), but found food containing 1% ethanol or more aversive (Korine et al. 2011). These findings suggest that in species naturally exposed to ethanol in their environment, low-level, ecologically relevant concentrations are unlikely to disrupt appetite, whereas adverse effects are more likely to occur only at higher doses.

Although the overall consumption of sucrose solution did not differ between *Nosema*-infected and control honeybees, the proportion of ethanol-spiked food consumed relative to total intake revealed a pattern. Infected individuals had 26% higher odds of choosing ethanol-containing solutions compared to control honeybees. Unlike in Mustard et al. (2019), where honeybees exhibit a general dose-dependent preference for moderate ethanol concentrations (1.25% to 2.5%), our control honeybees showed no general preference for ethanol, suggesting that increased ethanol intake is infection-dependent. One possible explanation for this pattern is self-medication. Honeybees are known to use environmental substances to mitigate infections (Gherman et al. 2014; Pusceddu et al. 2019). According to Abbott (2014), four criteria define self-medication: (1) active use of the substance by the host, (2) negative impact of the substance on the parasite, (3) increased host fitness when infected, and (4) cost to uninfected individuals. In our study, criterion (1) appears to be met, as infected honeybees increased their ethanol consumption actively. Criterion (4) is supported by previous research showing that, although honeybees are generally highly resilient to low doses of ethanol (Ostap-Chec et al. 2025a), its toxic effects can emerge with age even at occasional consumption (Ostap-Chec et al. 2024b), and it is harmful to winter bees even at low doses (Rasmussen et al. 2021). Although criteria (2) and (3) were not directly tested in the current experiment, our survival data provide preliminary conclusions.

Our survival analysis showed that higher ethanol consumption increased overall mortality, and honeybees on the 1% ethanol diet had a 26% higher mortality risk compared to those on the 0.5% ethanol diet. This highlights the potential costs of ethanol consumption. However, the interaction between infection status and diet revealed that the negative effect of 1% ethanol on survival was less pronounced in infected honeybees than in healthy controls. Indeed, in the absence of an interaction, infected honeybees given 1% ethanol would be expected to show ∼33% higher mortality than what was observed due to an additive effect of diet and infection status. This could be interpreted as an indication that ethanol consumption improves the fitness of infected individuals, thus partially fulfilling the third criterion for self-medication behavior. Whether infected foragers share ethanol-spiked food with nestmates remains unknown. If they do, this could represent a form of collective self-medication, a possibility that warrants further investigation. At the same time, whether ethanol directly affects the parasite remains unclear. In our study, spore loads did not differ significantly between the 0.5% and 1% ethanol diets, suggesting no stronger inhibitory effect on *Nosema* at the higher concentration. Yet, while spore counts reflect the intensity of infection, they do not fully capture parasite fitness. Therapeutic effects could still occur through changes in traits like transmission, infectivity, or spore viability—an issues requiring extensive further study.

Taken together, our results indicate that ethanol consumption may serve as a form of self-medication. However, since not all criteria for self-medication are fully met, alternative explanations should also be considered. One such explanation is that ethanol affects honeybee gut physiology rather than acting directly on *N. ceranae* spores. Since gut conditions strongly influence parasite development, even slight damage to the peritrophic matrix can accelerate infection (de Oliveira et al. 2023). Ethanol represents a complex dietary component, acting simultaneously as a toxin (Ostap-Chec et al. 2024b), behavioral modulator (Abramson et al. 2004a, 2005; Bozic et al. 2006; Maze et al. 2006; Ammons and Hunt 2008; Mustard et al. 2008; Mixson et al. 2010; Wright et al. 2012; Giannoni-Guzmán et al. 2014; Black et al. 2021; Ahmed et al. 2022) and a nutritional source (Pecsenye et al. 1994; Castro et al. 2012), influencing host physiology and immunity directly or modulating the gut microbiome. Positive physiological effects of low-dose ethanol intake have already been demonstrated in *Drosophila* and rodents (Justice et al. 2019; Diao et al. 2020; Chandler et al. 2022; Xuan et al. 2022). In honeybees, the gut microbiome plays a crucial role in immune responses, and supplements such as probiotics, phytochemicals, or natural compounds like *p*-coumaric acid have been shown to support gut function and reduce pathogen loads (Boncristiani et al. 2012; Mao et al. 2013; Geldert et al. 2021; Robino et al. 2024; Sbaghdi et al. 2024). It is therefore possible that ethanol ingestion may alleviate gut dysfunction or modulate microbiota balance in infected bees. However, as our study did not directly test these mechanisms, they remain speculative and warrant further investigation.

A third possible explanation is behavioral manipulation by *N. ceranae*. Such parasite-induced changes are well-documented (Hughes et al. 2012) and can enhance parasite fitness by affecting host behavior through the nervous system or neuromodulatory pathways (Adamo 2003; Libersat et al. 2009). *N. ceranae* is known to alter honeybee behavior. Infected workers spend more time working outside the hive (Dussaubat et al. 2013; Alaux et al. 2014; Naug 2014; Wolf et al. 2014) and begin foraging earlier than healthy individuals (Lecocq et al. 2016)—behaviors commonly interpreted as forms of social immunity. Infected honeybees also show increased walking activity and trophallaxis, potentially facilitating spore transmission (Lecocq et al. 2016). It is therefore conceivable that *N. ceranae* may also manipulate honeybee behavior to increase ethanol consumption, potentially gaining some yet unidentified benefit. However, this explanation has several limitations. Behavioral manipulation is evolutionarily costly and typically evolves only under specific conditions, such as low passive transmission rates or reduced parasite fecundity (Poulin 1994; Vickery and Poulin 2010), which do not apply to *N. ceranae*. This parasite transmits efficiently through multiple routes (Higes et al. 2008; Smith 2012; Sulborska et al. 2019) maintains high infection prevalence within colonies (Goblirsch 2018) and relies on a simple, energy-efficient life cycle confined to the gut (Gisder et al. 2011). Nonetheless, further research is needed to determine whether ethanol consumption benefits the parasite and whether it influences spore dynamics directly.

In summary, *N. ceranae* infection does not affect overall food intake in honeybees, but infected individuals consume significantly more ethanol-spiked solutions than healthy honeybees. Moreover, ethanol intake may partially improve the survival of infected honeybees despite its general toxicity to healthy individuals, suggesting a potential self-medication effect. However, spore load did not differ between ethanol concentrations, indicating that ethanol may not affect the parasite directly or that both doses have comparable effects. Alternatively, ethanol could influence gut physiology or immunity, possibly via modulation of the gut microbiota, or less likely, reflect parasite-induced manipulation. Nonetheless, our study is the first to demonstrate a prolonged preference for ethanol in honeybees, supporting the idea that ethanol is not just a dietary component but a behaviorally relevant substance. These findings suggest that honeybees can modulate their intake of naturally occurring compounds like ethanol in ways that may influence their health and resilience to infection, highlighting a more prominent role of ethanol in honeybee ecology than previously recognized.

## Data Availability

Analyses reported in this article can be reproduced using the data provided by Ostap-Chec et al. (2025c).
